# Synthesis of Fe-Al-Ti Based Intermetallics with the Use of Laser Engineered Net Shaping (LENS)

**DOI:** 10.3390/ma8052311

**Published:** 2015-04-29

**Authors:** Monika Kwiatkowska, Dariusz Zasada, Jerzy Bystrzycki, Marek Polański

**Affiliations:** Department of Advanced Materials and Technologies, Military University of Technology, 2 Kaliskiego Str., 00-908 Warsaw, Poland; E-Mails: monika.kwiatkowska@wat.edu.pl (M.K.); dariusz.zasada@wat.edu.pl (D.Z.)

**Keywords:** iron aluminides, LENS, laser cladding, rapid solidification alloying, microstructure, synthesis

## Abstract

The Laser Engineered Net Shaping (LENS) technique was combined with direct synthesis to fabricate L2_1_-ordered Fe-Al-Ti based intermetallic alloys. It was found that ternary Fe-Al-Ti alloys can be synthesized using the LENS technique from a feedstock composed of a pre-alloyed Fe-Al powder and elemental Ti powder. The obtained average compositions of the ternary alloys after the laser deposition and subsequent annealing were quite close to the nominal compositions, but the distributions of the elements in the annealed samples recorded over a large area were inhomogeneous. No traces of pure Ti were observed in the deposited alloys. Macroscopic cracking and porosity were observed in all investigated alloys. The amount of porosity in the samples was less than 1.2 vol. %. It seems that the porosity originates from the porous pre-alloyed Fe-Al powders. Single-phase (L2_1_), two-phase (L2_1_-C14) and multiphase (L2_1_-A2-C14) Fe-Al-Ti intermetallic alloys were obtained from the direct laser synthesis and annealing process. The most prominent feature of the ternary Fe-Al-Ti intermetallics synthesized by the LENS method is their fine-grained structure. The grain size is in the range of 3–5 μm, indicating grain refinement effect through the highly rapid cooling of the LENS process. The Fe-Al-Ti alloys synthesized by LENS and annealed at 1000 °C in the single-phase B2 region were prone to an essential grain growth. In contrast, the alloys annealed at 1000 °C in the two-phase L2_1_-C14 region exhibited almost constant grain size values after the high-temperature annealing.

## 1. Introduction

Fe_3_Al and FeAl-based iron aluminides are potential structural materials for high temperature applications in hostile environments. However, the insufficient strength, creep resistance and ductility of these aluminides at room temperature have been identified as obstacles for their practical use [1]. Palm [2] presented different strategies for strengthening Fe-Al-based alloys at high temperatures, including solid solution hardening, strengthening by coherent or incoherent precipitates and increasing the long-range order. In this respect, the Fe-Al-Ti system seems to be very interesting because it offers the possibility of applying these various strengthening mechanisms. It has been reported that Ti considerably increases the critical temperature for the D0_3_-B2 transition reordering from 552 °C for stoichiometric Fe_3_Al with the D0_3_ structure to approximately 1215 °C for the stoichiometric Fe_2_TiAl Heusler phase with the L2_1_ structure, which is the ternary equivalent of the binary D0_3_ structure [3]. In addition, for certain compositions with a low Ti content, there is a miscibility gap between the A2 and L2_1_ structures where coherent precipitates can be formed. There is also the possibility for the precipitation of second phases, such as a Laves phase in equilibrium with the L2_1_ phase or the cubic Mn_23_Th_6_-type phase τ_2_ [4,5].

The mechanical behavior of ternary Fe-Al-Ti intermetallic alloys has recently been studied. The beneficial influence of Ti on the strength, creep resistance and oxidation behavior at elevated temperatures has been demonstrated [6,7,8,9,10,11]. Compared to other iron aluminides, the ternary Fe-Al-Ti intermetallic alloys exhibit a higher yield stress and creep resistance at temperatures up to 800 °C. Palm and Sauthoff [7] reported that the properties of ternary Fe-Al-Ti alloys are controlled by not only the Al and/or Ti content but also by the phase formation and corresponding microstructure. In fact, grain refinement in the L2_1_-ordered Fe-Al-Ti alloys by TiB_2_ precipitates results in the lowering of the brittle-to-ductile transition temperature (BDTT) [9,10]. Therefore, there is some potential to lower the BDTT by reducing the grain size of ternary Fe-Al-Ti alloys, which usually have a coarse-grained structure after conventional casting.

In recent years, the direct manufacturing of metallic components using the Laser Engineered Net Shaping (LENS) technique has been shown to be a promising rapid additive manufacturing technology that could significantly reduce time between an initial concept and a finished component. The LENS is a solid-freeform fabrication method that can be used to manufacture bulk metallic parts directly from CAD files. During the laser forming, a powder is fed into a melt pool that is produced by a sharply focused laser beam. Parts are built in a layer-by-layer fashion by rastering the laser and powder source across the substrate [12]. Various benefits are exhibited by the additive, tool-less character of this process, especially in the manufacture of highly shaped parts made of expensive and difficult-to-form high performance engineering materials. The LENS technique is especially recommended for producing components made of stainless steels, Ni-based alloys, Ti-based alloys, refractory metals and intermetallics that are difficult or impossible to produce through conventional metal forming techniques [12]. The LENS technique offers many unique processing advantages, such as the ability to retain rapid solidification effects and to design composition gradients in near-net shape components. Because the LENS technique uses a powder feedstock with multiple hoppers, it also allows mixing elemental powders for the *in situ* formation of alloys.

There have been a limited number of studies on the *in situ* direct laser deposition of alloys from elemental powder blends [13,14,15,16]. Collins *et al.* [14] and Schwendner *et al.* [15] used this approach to study Ti-Mo-Nb-Al-Si, Ti-Cr-Nb-Al-Si, Ti-Cr, and Ti-Nb alloys fabricated using the LENS technique. Their results reveal that the thermodynamic enthalpy of mixing of the constituent elements is the most important factor determining the microstructure and compositional homogeneity of these alloys. Negative enthalpy of mixing results in a more homogeneous intermixing in the melt pool and a rapid solidification rate. In contrast, positive enthalpy of mixing results in poor intermixing, an inhomogeneous alloy, and a slower rate of solidification. In addition to the enthalpy of mixing, there are other important LENS processing parameters that contribute to the total energy input, such as the laser power, traverse speed, hatch width and layer spacing. Therefore, all of these factors should be correlated by an energy density term [14,15]. If the energy density is sufficiently large, *i.e.*, greater than a certain critical value, then the as-received component is characterized by mechanical properties similar to that of a conventionally produced wrought alloy of the same chemical composition.

The present work explores the possibility of depositing ternary Fe-Al-Ti intermetallics *in situ* from a feedstock composed of pre-alloyed Fe_3_Al and FeAl intermetallic powders and elemental titanium powder using the LENS process. The results of our preliminary studies show that the manufacture of components using the LENS method combined with direct alloy synthesis is a very promising freeform technique that has the significant advantage of rapid cooling (up to 10^4^ K/s). Such conditions should lead to a significant non-equilibrium solute-trapping effect, thereby avoiding component segregation and relieving solubility limitations. Consequently, a strength enhancement effect due to grain refinement and the formation of solid solutions is achieved [17,18,19]. The laser forming of components combined with direct alloy synthesis is a new and very promising direction in the additive manufacturing of high-performance components that contain an optimized combination of different materials, such as alloys, composites and functionally graded materials. Furthermore, we have found that LENS processing combined with direct alloy synthesis can be a very promising manufacturing technique for the rapid production of high melting alloys, including intermetallics and multicomponent high entropy alloys [20]. The other interesting aspect of this approach is the possibility of combinatorial synthesis using LENS of compositionally graded bulk libraries for rapid investigations of metallic functional and structural materials [21,22,23,24]. The combinatorial approach based on the direct laser deposition of compositionally graded alloys using the LENS technique was demonstrated by Nag *et al.* [23], who revealed that the combinatorial synthesis of Ti-based biomaterials for orthopedic and dental prostheses by LENS is a very attractive and efficient method for the rapid assessment of changes in the microstructure and mechanical properties as a function of composition. Most recently, Polanski *et al.* [24] showed that gradient and discrete multisample alloy libraries can be successfully manufactured using LENS technology. Binary Ti-Fe and ternary Ti-Fe-Ni alloy libraries were fabricated either from elemental powders delivered from different powder feeders or from pre-blended powder mixtures.

## 2. Results and Discussion

**Figure 1:**
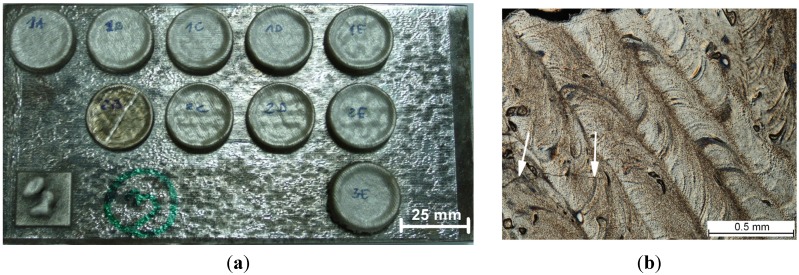
(**a**) Fe-Al-Ti samples deposited by the LENS method; and (**b**) layered structure of the deposited sample no. 1 (view from the top of the sample). Arrows indicate cracks.

**Figure 2:**
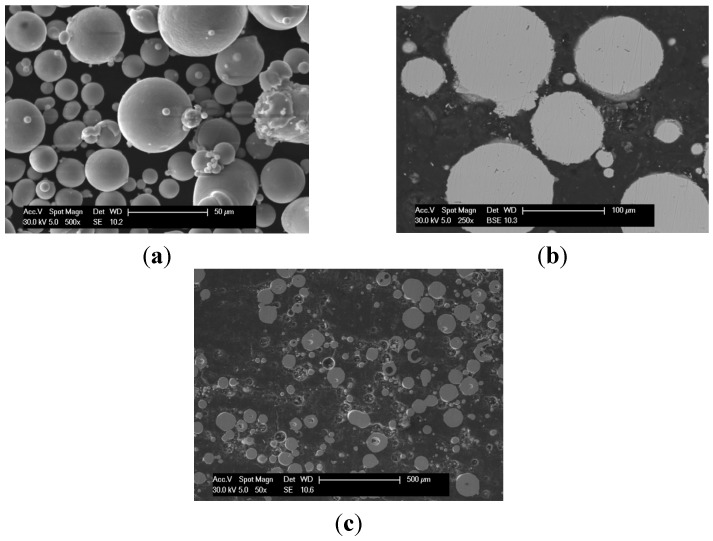
Morphology of the initial gas atomized Fe-40Al (at. %) powder: (**a**) SEM SE image showing spherical particles; (**b**) SEM BSE image of the cross-section of the particles that reveals their chemical homogeneity; and (**c**) voids in the volume of the particles.

A photograph showing examples of cylindrical samples produced using the LENS method is shown in Figure 1a. It is observed that fully dense bulk ternary Fe-Al-Ti intermetallic samples can be produced using LENS combined with direct alloy synthesis from pre-alloyed FeAl and elemental Ti powders. An inspection of the deposited sample geometry indicates that the diameters produced were within 24.48–25.65 mm of the specified diameter. However, macroscopic cracking caused some samples to delaminate from the substrate, and disintegration of the samples due to high thermal stresses was occasionally observed. Macroscopic cracks were also observed on the upper surface of the deposited samples. Figure 1b shows the layered structure of the LENS fabricated sample no. 1 (view from the top), with a crack that crosses the deposited layers. Most recently, Karczewski *et al.* [25] reported that residual stresses in iron aluminides fabricated using LENS are very close to their yield strength and have a compressive character in the center of the specimen and sometimes a tensile character near the upper surface. The post-processing heat treatment essentially reduces the level of the resulting residual stresses. In addition, spherical pores within the deposited samples were observed in all of the alloys. Defects that typically occur during layer-by-layer additive manufacturing, such as a lack of fusion at the interlayer boundaries and interlayer porosity, were not observed. A summary of the measured porosities of the deposited alloys is provided in Table 1. In general the amount of porosity in the deposits is less than that in the starting Fe-Al powders. Sample no. 1 exhibited the lowest porosity of 0.2 vol. %, while sample no. 3 had the highest, 1.2 vol. %. Based on an analysis of the pore morphology, it can be assumed that the presence of the porosity originates from the porous pre-alloyed Fe-Al powders (Figure 2c). The LENS solidification process is so rapid that pores introduced in the melt pool can be trapped simply because the very rapid cooling (~10^3^–10^4^ K/s) does not allow the gas to escape. Our most recent findings have revealed that heating the substrate during the laser deposition process and increasing the laser power essentially decrease the cooling rate and reduce residual stresses. Changing the substrate material to an alloy with a composition closer to the composition of the deposited ternary Fe-Al-Ti alloys should also lower thermal stresses generated due to the difference between the thermal expansion coefficients of the two materials. Investigations in this area are currently in progress in our group.

**Table 1:** Nominal and obtained chemical compositions of the Fe-Al-Ti alloys and the phase content, grain size, hardness and porosity after laser deposition and annealing at 1000 °C for 20 h with furnace cooling.

Alloy no.	Alloy condition	Chemical composition (at. %)			Constituent phases	Grain size (μm)	Vickers hardness HV_0.1_	Porosity (vol. %)
		Fe	Al	Ti				
1	Nominal	69.2	23.3	7.5	L2_1_ + A2 + C14	-	-	-
	As-deposited	69.7 ± 0.2	21.8 ± 0.1	8.5 ± 0.1	L2_1_ + C14	4.3 ± 2.9	570 ± 150	0.2
	Annealed	69.3 ± 0.3	22.2 ± 0.1	8.5 ± 0.1	L2_1_ + A2 + C14	18.3 ± 15.3	535 ± 22	-
2	Nominal	59.8	20.2	20.0	L2_1_ + C14	-	-	-
	As-deposited	60.1 ± 0.3	18.1 ± 0.2	21.8 ± 0.2	L2_1_ + C14	~6 * ~5 *	814 ± 51 903 ± 40	0.5
	Annealed	60.0 ± 0.3	19.1 ± 0.2	20.9 ± 0.1	L2_1_ + C14	~9 * ~3 *	645 ± 19 663 ± 54	-
3	Nominal	54.0	36.0	10.0	L2_1_	-	-	-
	As-deposited	53.2 ± 0.2	36.2 ± 0.2	10.6 ± 0.1	L2_1_	3.0 ± 1.9	428 ± 28	1.2
	Annealed	55.2 ± 0.2	34.6 ± 0.2	10.2 ± 0.1	L2_1_	40 ± 17 ** 188 ± 69 **	347 ± 11	-
4	Nominal	48.0	32.0	20.0	L2_1_ + C14 + τ_2_	-	-	-
	As-deposited	47.5 ± 0.2	30.6 ± 0.3	21.9 ± 0.2	L2_1_ C14	3.1 ± 2.2 0.8 ± 0.4	594 ± 29 802 ± 57	0.6
	Annealed	48.6 ± 0.3	29.3 ± 0.3	22.1 ± 0.2	L2_1_ C14	4.5 ± 3.0 1.3 ± 1.0	538 ± 40 883 ± 55	-

Note: * The grain size distributions of both phases after laser deposition and annealing were inhomogeneous. The estimated sizes of the phases were measured by OIM; ** the sizes of the L2_1_ grains exhibited a bimodal distribution after the high-temperature annealing.

**Figure 3:**
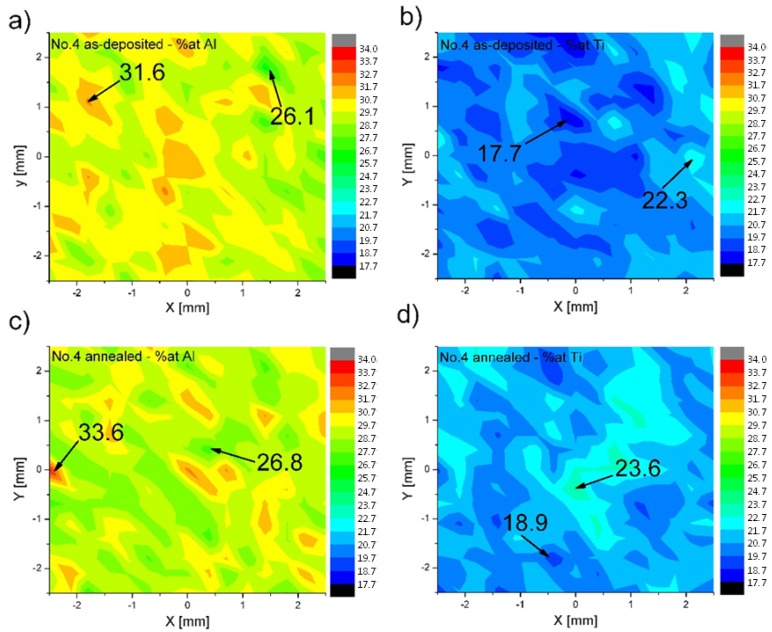
XRF macro-maps showing the distributions of Al and Ti in sample no. 4 after the LENS and subsequent annealing at 1000 °C for 20 h with furnace cooling: (**a**) Al—LENS; (**b**) Ti—LENS; (**c**) Al—LENS and annealing; (**d**) Ti—LENS and annealing.

The compositions of the ternary alloys measured by wavelength dispersive X-ray fluorescence (XRF) spectroscopy after the laser deposition and subsequent annealing are listed in Table 1. The obtained average compositions are quite close to the nominal compositions. For samples no. 1 and 3, an average difference of approximately 1 at. % between Al and Ti is observed. The greatest variation is observed in samples no. 2 and 4, which exhibited a loss of Al and an excess of Ti of approximately 2 at. %, respectively. This result suggests that there are some difficulties in obtaining homogenous melt pools during the laser deposition process. The XRF macro-maps recorded over large areas (5 × 5 mm) of the alloys revealed that the distributions of elements in the deposited and subsequently annealed samples are inhomogeneous. Figure 3 presents the distribution of Al and Ti in sample no. 4, with high Ti and Al contents, after the LENS deposition and subsequent annealing. Even after the high temperature annealing, there are still some regions in the sample where the amounts of Al and Ti are essentially different from the average composition. No pure Ti was observed in the deposited alloys. It would seem that a longer annealing time is needed to obtain better chemical homogeneity. For example, Krein and Palm [8] annealed L2_1_-ordered Fe-Al-Ti alloys at 1000 °C for 500 h under an argon atmosphere to obtain a homogeneous distribution of elements. To avoid thermal stresses and cracking, the alloys were subsequently furnace-cooled to room temperature. However, as inhomogeneities in our samples are in the scale of millimeters, it is doubtful that they can be leveled out within reasonable times during heat treatment. Therefore, the optimization of the processing parameters of the direct alloy synthesis is required to obtain a better chemical homogeneity. Zhang *et al.* [16] observed during the laser powder micro-deposition of compositional graded Ti-Cr alloy that the addition of Cr requires a higher laser energy for the complete melting and mixing of the particles due to the higher melting temperature of Cr than Ti and its lower absorptivity, which is proportional to the square root of the electrical resistivity of the material. The melting temperature of Ti (~1670 °C) is higher than those of both the pre-alloyed Fe25Al at. % (~1420 °C) and Fe40Al at. % (~1520 °C) powders used in the synthesis of the ternary Fe-Al-Ti alloys. There is also an essential difference in the electrical resistivity (and thereby also the absorptivity) between Ti (5.5 × 10^−7^ Ωm) and Fe25-40Al at. % (10 ÷ 15 × 10^−7^ Ωm [26]). Collins *et al.* [14] suggested that the enthalpy of mixing should also be used as a practical guideline for selecting alloy compositions and processing parameters for the direct laser synthesis of alloys from elemental powders, e.g., some amount of the pre-alloyed FeAl powder can be used as an elemental Fe and Al powder blend to generate additional heat in the melt pool. However, the problem of the soft Al powder must be considered to avoid damage to the powder feed wheel in the LENS machine. Taking into account the above, it appears that the optimization of the LENS processing parameters, in particular the laser energy, scanning speed and substrate temperature, which affect the cooling rate, the temperature gradient and the diffusion/mixing time of elements during the synthesis, is the best way to improve the chemical homogeneity of the investigated ternary Fe-Al-Ti alloys.

**Figure 4:**
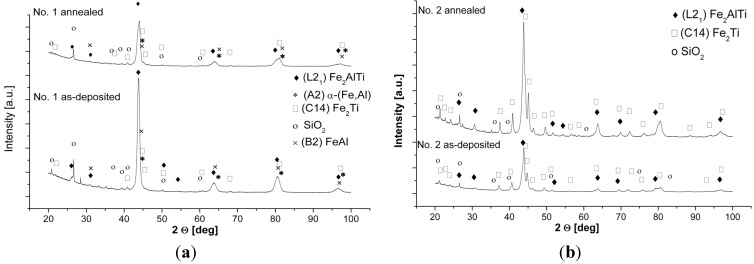

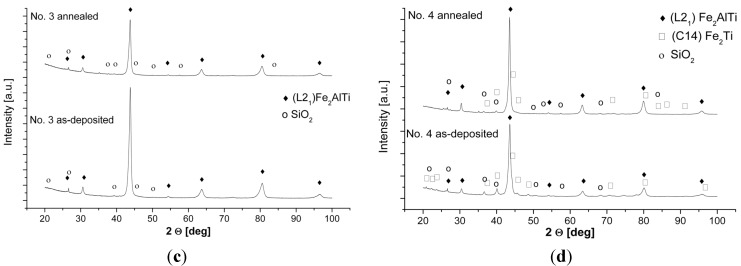
XRD patterns of the investigated alloys after laser deposition and annealing at 1000 °C for 20 h with furnace cooling: (**a**) sample no. 1; (**b**) sample no. 2; (**c**) sample no. 3; (**d**) sample no. 4.

**Figure 5:**
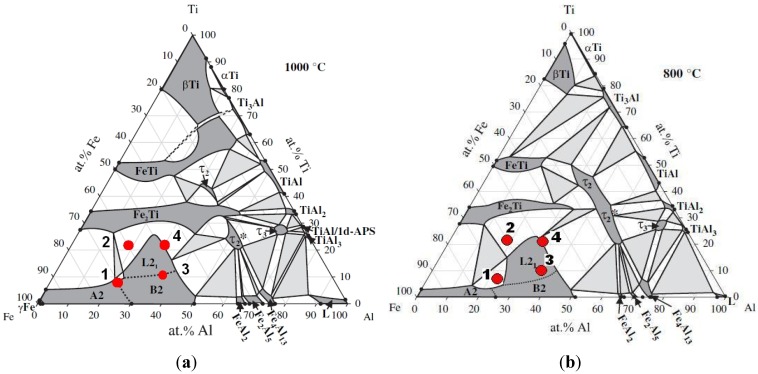
Isothermal sections of the Fe-Al-Ti system at 1000 °C (**a**) and 800 °C (**b**) with the compositions of the investigated alloys [5].

The XRD patterns of the as-deposited and subsequently annealed samples of the investigated alloys are shown in Figure 4. A summary of the identification of the constituent phases combined with the microstructural characterization of grain size, microhardness and porosity is provided in Table 1. The respective phase structures of the obtained alloys are shown in the isothermal sections of the ternary Fe-Al-Ti system at 1000 °C and 800 °C in Figure 5 [5].

**Figure 6:**
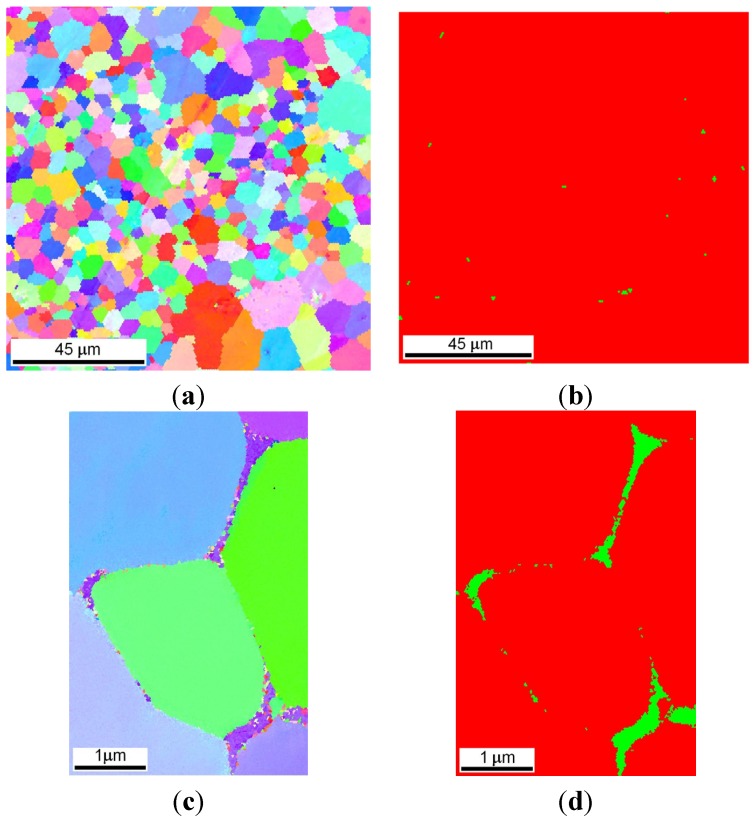

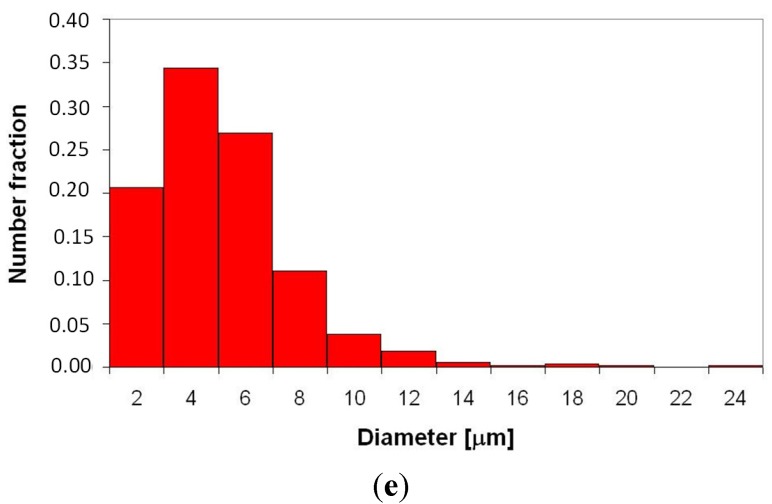
Microstructural characterization with OIM of sample no. 1 after laser deposition: (**a**) EBSD inverse pole figure map; (**b**) phase composition map: red color denotes the L2_1_ Fe_2_TiAl Heusler phase, green color denotes the—C14 Fe_2_Ti Laves phase; (**c**,**d**) high magnification of OIM maps showing the distribution of the C14 Laves phase (green) in the L2_1_ matrix (red color); (**e**) the grain size distribution of the L2_1_ Fe_2_TiAl phase.

After the LENS deposition, sample no. 1 presents an L2_1_ Fe_2_(Fe,Ti)Al Heusler phase matrix with some traces of the C14 (Fe,Al)_2_Ti Laves phase (Figure 4a and Figure 6). Additionally, a small amount of an unidentified phase was found in this alloy after the laser deposition and annealing. There are also three peaks that are derived from the contamination of the alloy by C and O. These elements were determined only qualitatively using XRF and EDS and could be introduced into the material during the sample preparation. An inspection of the isothermal sections of the Fe-Al-Ti system shown in Figure 5 indicates that sample no. 1 should exhibit the B2 (Fe,Ti)Al phase matrix after laser deposition. However, the reheating caused by successive laser tracks leads to the continuous annealing of the deposited layers over a wide temperature range. Additionally, a great chemical inhomogeneity in the scale of millimeters leads to the occurrence of a Laves phase in the areas which are rich in Ti. Furthermore, the BSE/EDS analysis of the alloy revealed “dark and white” microregions after laser deposition. The white regions are Al-rich (21.2 ± 0.6 at. %) and Ti-rich (11.6 ± 0.5 at. %) and Fe-deficient (67.2 ± 0.9 at. %) compared to the dark regions, which are rich in Fe (72.1 ± 0.4 at. %) and Ti-deficient (7.3 ± 0.3 at. %). Taking into consideration the large inhomogeneity of the alloy and its reheating, it is possible that there are also regions with the B2 FeAl phase and/or a disordered A2 α (Fe,Al) solid solution. However, it is not possible to distinguish between these phases on the basis of the obtained X-ray diffraction patterns. The diffraction peaks from both the B2 and A2 phases are marked in Figure 4a. Nevertheless, the OIM phase analysis carried out in the selected areas confirmed the dominant presence of the L2_1_ and C14 Laves phases after the laser deposition (Figure 6). The C14 Laves phase only occurs as small precipitates along the grain boundaries, and its fraction is approximately 2 vol. % (Figure 6a–d). The L2_1_ matrix of this alloy has an equiaxed grain structure with an average grain size of 4 μm and a uniform distribution of grain sizes in the volume of the deposited sample (Figure 6a,e).

**Figure 7:**
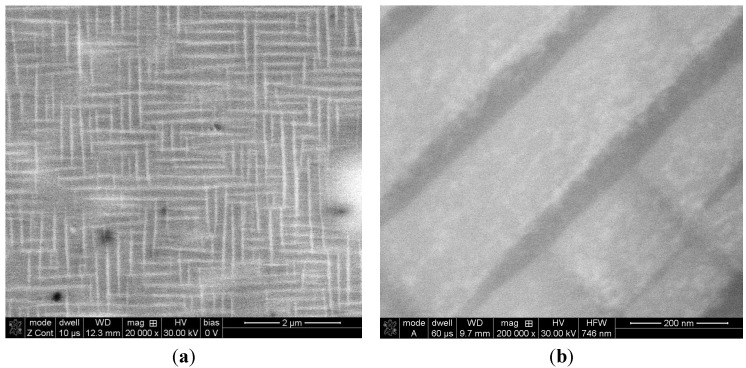
(**a**) SEM BSE image showing the coherent two-phase microstructure of sample no. 1 after annealing; (**b**) STEM image taken from a lamella that was cut from the area shown in Figure 7a. Nearly needle-like in cross-section and nanometric in diameter Fe-rich α-(Fe,Al) precipitates are embedded in an L2_1_-ordered phase.

After annealing at 1000 °C for 20 h in an argon atmosphere with furnace cooling, sample no. 1 exhibited a coherent microstructure of the ordered L2_1_-phase and some amounts of the disordered A2 phase and the C14 Laves phase, which predominantly precipitated along the grain boundaries. The broadening of all the diffraction peaks after annealing can be attributed to the presence of nanometric (in diameter) precipitates embedded into a grayish phase (Figure 4a and Figure 7). Considering the isothermal sections of the Fe-Al-Ti system shown in Figure 5, sample no. 1 should exhibit the B2 (Fe,Ti) Al phase after annealing at 1000 °C. However, the slow furnace cooling caused the B2 phase to decompose into the L2_1_ and A2 phases. Additionally, a large chemical inhomogeneity on the scale of millimeters is probably responsible for the occurrence of the Laves phase. Figure 7a presents the coherent two-phase microstructure in the STEM DF mode, which clearly shows black, nearly needle-like in cross-section and nanometric in diameter, Fe-rich α-(Fe,Al) precipitates embedded in a grayish L2_1_-ordered phase. These α-(Fe,Al) precipitates with a disordered A2 structure are quite uniformly distributed within the L2_1_ matrix. The OIM microstructural characterization of this alloy revealed that an essential grain growth occurs in the alloy while annealing at 1000 °C for 20 h. The average grain size of the L2_1_/A2 matrix was 18 μm after annealing, compared to 4 μm after laser deposition (Table 1). The majority of the grain boundaries among the L2_1_/A2 matrix grains belong to HABs, with a high density of LABs forming sub-grain structures. It is worth noting that sample no. 1 was in the single-phase B2 region (Figure 5a) while annealing at 1000 °C, making it more prone to grain growth. At lower temperatures, the nanometric α-(Fe,Al) precipitates should effectively restrain the grain growth and lead to a finer grain microstructure. The average microhardness of the annealed samples was slightly less than that of the samples deposited with LENS (Table 1). The annealing process leads to a reduction of the residual stresses generated in the sample during laser deposition. Therefore, the high hardness of this alloy after annealing can be attributed to a hardening effect that results from the presence of coherent precipitates in the matrix and also to the presence of hard Laves phase particles.

**Figure 8:**
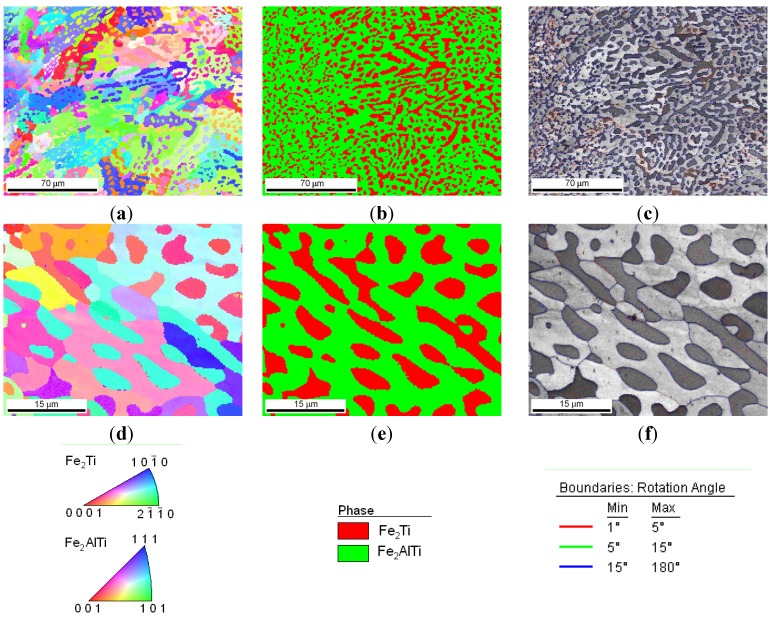
The microstructural characterization with OIM of sample no. 2 after laser deposition and annealing: (**a**) orientation map; (**b**) phase composition map: red color denotes the L2_1_ Fe_2_TiAl Heusler phase, and green color denotes the C14 Fe_2_Ti Laves phase; (**c**) boundary map; (**d**,**e**,**f**) high magnifications of maps from figures (**a**–**c**).

**Figure 9:**
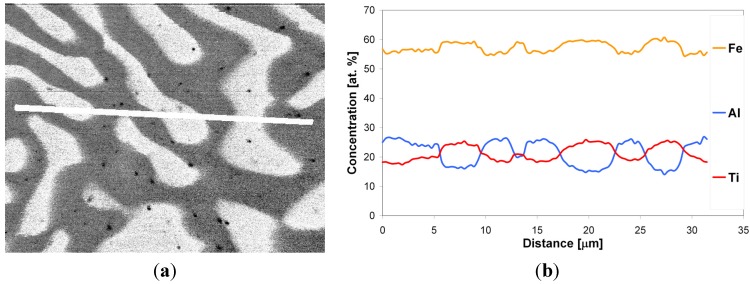
(**a**) BSE image of sample no. 2 after laser deposition and annealing at 1000 °C for 20 h; (**b**) linear EDS analysis of Fe, Al and Ti along the line in figure (**a**).

After the LENS deposition, sample no. 2 exhibited two phases, the L2_1_ Fe_2_(Fe,Ti)Al and the C14 (Fe,Al)_2_Ti Laves phases, as shown in the XRD pattern in Figure 4b. This alloy contains the highest amount of Ti, which is why it is notable that there were no traces of pure titanium in this sample. An analogous two-phase composition, *i.e.*, L2_1_/C14 Laves phases, was observed after laser deposition and subsequent annealing at 1000 °C for 20 h with furnace cooling. The phase composition of this alloy after laser deposition and additional annealing is consistent with the isothermal sections of the Fe-Al-Ti system in Figure 5. The increased intensities of the diffraction peaks of both phases after the heat treatment are clearly visible in Figure 4b. This alloy, after laser deposition and annealing, also exhibited some contamination and other unidentified phases, similar to sample no. 1. The OIM microstructural characterization reveals that spherical Laves phase precipitates are distributed in the L21 phase matrix after the LENS deposition and annealing (Figure 8). The volume fraction of the C14 Laves phase after laser deposition is approximately 31%, and it remains essentially constant after annealing (33 vol. %). The grain size distributions of both phases after laser deposition and annealing at 1000 °C were inhomogeneous. The OIM estimated sizes of both phases are very small, ~5 μm, and the sizes remain almost constant after the high temperature annealing (Table 1). This behavior can be attributed to the effect of the second phase because this alloy, while annealing at 1000 °C, was in the two-phase L2_1_-C14 region (Figure 5a). The Laves phase supplied resistance to the movement of the grain boundaries, as was observed in the Fe_3_Al alloy with NbC and Fe_3_AlZr second phases [27]. Figure 9 presents a micrograph of the annealed sample collected from the BSE detector, which reveals that there are two chemically distinguishable regions. Light oval precipitates are embedded in the gray matrix. An EDS line scan revealed that the oval precipitates are rich in Ti and Fe and are most likely the C14 Laves phase. The smaller amount of Fe and Ti in the oval precipitates can result from the fact that the EDS signal can overlap phases underneath the surface (not visible), *i.e.*, the L2_1_ phase in our case. The OIM measurements of the disorientation angle between grains in the L2_1_ matrix revealed that the majority of the grain boundaries belong to HABs. The same situation applies to the case of the interphase Laves/L2_1_ phase boundaries, *i.e.*, almost all of the measured disorientations are high angle disorientations. A high density of low angle (<5°) disorientations that form sub-grain structures were observed in both phases after laser deposition (not shown here, see Reference [17]). This result indicates the presence of a strong hardening effect in these phases after the laser deposition process and is in good agreement with the considerable hardness of the spherical Laves phase precipitates (903 HV0.1) and the L2_1_ matrix (814 HV0.1) (Table 1). After annealing, the average microhardness of both phases is approximately 650 HV0.1, which is considerably lower than that of the as-deposited sample. This is consistent with the lower density of low angle (<5°) disorientations within the interior of both phases after annealing (Figure 8c,f).

**Figure 10:**
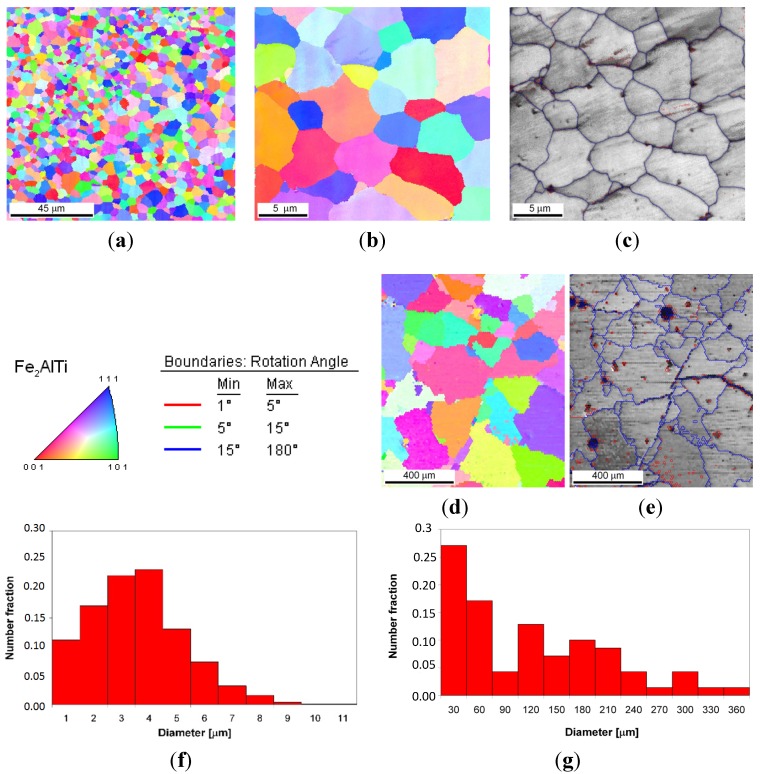
The OIM microstructural characterization of sample no. 3: (**a**,**b**) orientation and (**c**) boundary maps after laser deposition; (**d**) orientation and (**e**) boundary maps after laser deposition and annealing; (**f**) grain size distribution of L2_1_ Fe_2_TiAl phase; (**g**) grain size distribution of C14 Fe_2_Ti Laves phase. Color coded stereographic triangle is provided for orientation maps (**a**,**b**,**d**) and grain boundary rotation angle scale for grain boundary representation (**e**).

Figure 4c presents the XRD patterns of sample no. 3 that were obtained after the LENS deposition and subsequent annealing at 1000 °C for 20 h. The XRD patterns only reveal the presence of L2_1_ Fe_2_ (Fe,Ti) Al, and this is in accordance with the isothermal sections of the Fe-Al-Ti system in Figure 5, taking into account the reheating cycle caused by successive laser tracks. Again, there are no traces of Ti, only some unknown contamination, similar to the previous alloys. The OIM investigations of sample no. 3 after laser deposition confirm that it exhibits the L2_1_ phase and an equiaxed grain structure with an average grain size of 3 μm and a uniform distribution of grain sizes, as shown in Figure 10a,b,f. Similar to samples no. 1 and 2, almost all of the grain boundaries among the L2_1_ phase grains are HABs (~80%). A significant number of small disorientations within the interior of the L2_1_ grains were observed (Figure 10c). The most prominent feature of sample no. 3, which was annealed at 1000 °C for 20 h with furnace cooling, is an essential grain growth (Figure 10d,e,g). The sizes of the L2_1_ grains exhibited a bimodal distribution after the high-temperature annealing (Figure 10g). A considerable number of small disorientations within the interior of the small and large grains were observed (Figure 10c). Note that the annealing of sample no. 3 was performed at a temperature above the critical temperature for the L21/B2 transition (*i.e.*, in single phase B2 region), which is approximately 900 °C for this composition [3]. An abnormal grain growth in iron aluminide was also reported in References [27,28] after-high temperature annealing for a long time. The deviation in the composition of sample no. 3 from the stoichiometric composition for L2_1_ Fe_2_TiAl leads to an increase in the number of constitutional point defects, which are very mobile at high temperatures and most likely contribute to the grain growth [29]. Obviously, the constitutional point defects should also lead to a strong hardening effect, which corresponds to partial atomic disorder. However, note that the average microhardness of the L2_1_ phase alloy was approximately 395 HV_0.1_ after the laser deposition process and that it decreased to 340 HV_0.1_ after annealing. This value is lower than that of the previous two samples (no. 1 and 2) with the L2_1_ phase and also than the results obtained by Palm and Sauthoff [7], who studied the deformation behavior in single-phase L2_1_ and two-phase L2_1_/C14 Lave phase Fe-Al-Ti alloys. The authors observed that the lowest hardness is exhibited by the heat-treated single-phase L2_1_ Fe_2_(Fe,Ti)Al alloys in comparison to the single Laves phase and the two-phase L2_1_/Laves Fe-Al-Ti alloys. The high hardness was explained through deviations in the compositions of the alloys from the stoichiometric composition for the L2_1_ Fe_2_TiAl alloy, which increases the concentration of constitutional point defects. This hardening effect was evidently diminished when the temperature was increased above 800 °C. In contrast, Zho *et al.* [30] did not observe any variations in the hardness of the single-phase L2_1_ Fe-25Al (at. %) alloy that contained 0, 5, 10 and 15 at. % of Ti. More research is required to explain this opposite hardening behavior of non-stoichiometric, single-phase L2_1_ Fe-Al-Ti alloys.

**Figure 11:**
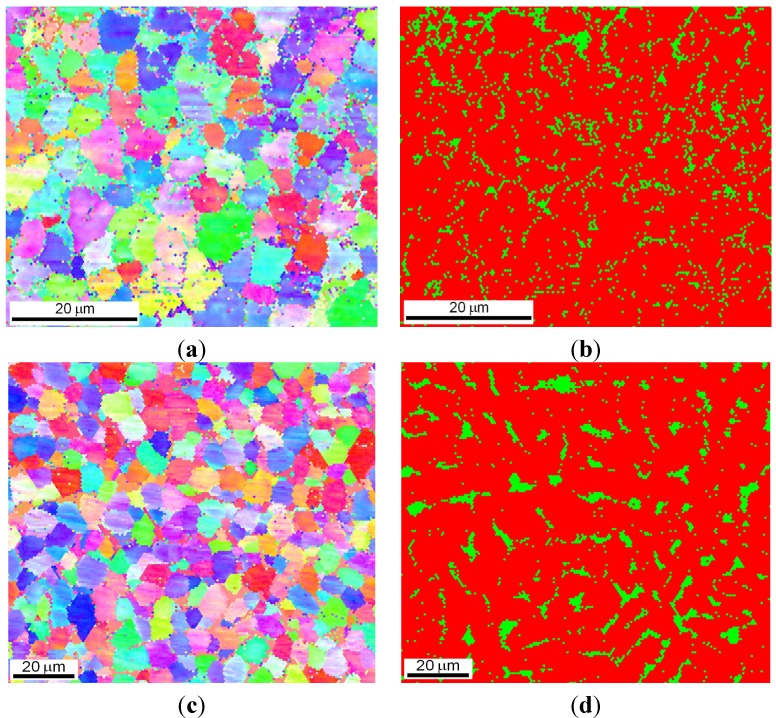
Microstructural characterization with OIM of sample no. 4 after laser deposition (**a**,**b**) and additional annealing (**c**,**d**); orientation maps (**a**,**c**) and phase composition maps (**b**,**d**): red color denotes the L2_1_ Fe_2_TiAl Heusler phase, and green color denotes the C14 Fe_2_Ti Laves phase.

**Figure 12:**
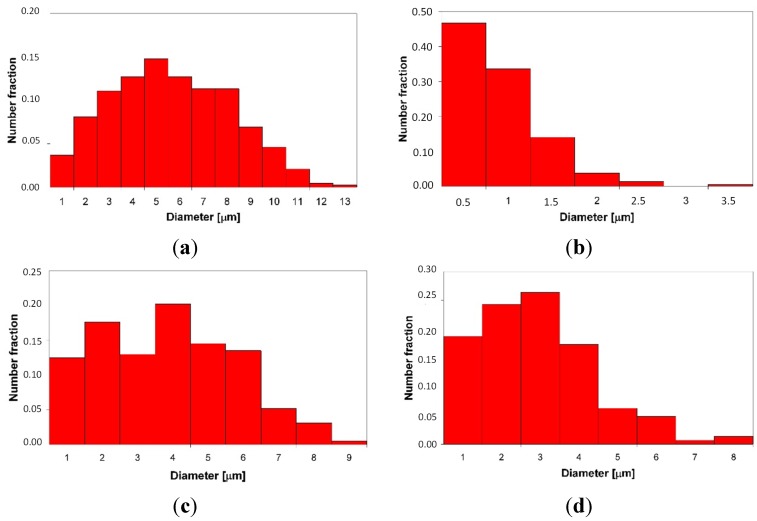
The grain size distribution of the L2_1_ Fe_2_TiAl phase (**a**,**c**); and C14 Fe_2_Ti Laves phase (**b**,**d**) after laser deposition (**a**,**b**) and additional annealing (**c**,**d**).

After the LENS deposition, sample no. 4 exhibited two phases, the L2_1_ Fe_2_ (Fe,Ti) Al and the C14 (Fe,Al)_2_Ti Laves phases, as shown in the XRD pattern in Figure 4d. An analogous two-phase composition, *i.e.*, the L2_1_/C14 Laves phases, was observed after laser deposition and subsequent annealing at 1000 °C for 20 h with furnace cooling. No XRD peaks from the cubic Mn_23_Th_6_-type τ_2_ phase were detected, as they occur at much higher Al contents (Figure 5). The OIM microstructural characterization of this alloy revealed that it has an equiaxed grain structure. The C14 Laves phase only occurs as small precipitates along the grain boundaries (Figure 11). This microstructure is very stable during high-temperature heat treatment. After annealing, the average grain size of the L2_1_ matrix was ~5 μm, compared to ~3 μm after laser deposition (Table 1). Furthermore, a uniform distribution of L21 grain sizes in the volumes of the deposited and annealed samples was observed (Figure 12a,c). The distribution of the C14 Laves phase after laser deposition is quite different than that of the heat-treated material (Figure 12b,d), indicating that the annealing process leads to the coalescence of the C14 Laves phase precipitates. Note that this alloy, while annealing at 1000 °C, was in a two-phase L2_1_-C14 region (Figure 5a). Like in sample no. 2, the Laves phase precipitates effectively restrain the movement of grain boundaries during high-temperature annealing. The differences between the microhardnesses of both the L2_1_ and C14 phases after laser deposition and annealing were in the range of statistical error (Table 1).

The most prominent feature of the ternary L2_1_–ordered Fe-Al-Ti intermetallics synthesized by LENS is their fine-grained structure after laser deposition. The grain size was 3–5 μm, which demonstrates the grain refinement effect through the very rapid cooling (10^3^–10^4^ K/s) of the LENS process. Fine grain sizes of 5–10 μm were previously obtained in B2 FeAl intermetallic ribbons produced by selective laser melting (SLM) by Song *et al.* [31]. The grain sizes on the order of 3–5 μm were also found in binary Fe-Al and ternary Fe-Al-Ti intermetallic ribbons produced by rapid solidification, with cooling rates of up to approx. 10^6^ K/s [32]. The fine-grained structure of iron aluminides is of interest in terms of increasing ductility and strength [1] as well as lowering the brittle-to-ductile transition temperature, as concluded in References. [7,10].

## 3. Experimental Section

Single-phase, two-phase and multiphase Fe-Al-Ti intermetallic alloys were produced using the LENS technique from pure Ti (Grade 2) powder obtained from TLS-Technik (Germany), pre-alloyed Fe-25Al at. % powder from Sandvik Osprey (Sweden) and Fe-40Al at. % powder from LERMPS (France). Pre-alloyed Fe-Al powders were used because the soft Al powder could damage the powder feed wheel. The powders were produced using the gas (argon) atomization method and exhibited a spherical particle shape with a size of 40–150 microns (Figure 2a). The Fe-25Al and Fe-40Al powders exhibited chemical homogeneity (Figure 2b) and an internal spherical porosity of 1–3 vol. % (Figure 2c). The voids in the Fe-Al particles were most likely produced by the entrapment of the atomizing gas (e.g., argon) during the formation of the initial droplet. A small amount of Si (max. 1 at. %) was found in the Fe-25Al powder.

The deposition of the Fe-Al-Ti alloys was conducted using a LENS MR7 system that was equipped with 4 powder feeders and a 500 W fiber laser, which had a minimum beam diameter of 200 μm at a central emission wavelength of 1070 nm. This is the first such LENS system manufactured by Optomec Inc. (US) [12] and is installed in the Military University of Technology in Poland [33]. Four alloys were investigated in detail, referred to as no. 1–4. The nominal compositions of the laser-deposited alloys are provided in Table 1. The desired compositions of the ternary Fe-Al-Ti alloys were achieved by using different blends of the pre-alloyed Fe-Al and Ti powders and by adjusting the flow rate of the powder feeders. The compositions of the single-phase, two-phase and multiphase Fe-Al-Ti alloys were selected based on their mechanical and corrosion properties for high-temperature applications, the available literature data about the Fe-Al-Ti system [3,4,5] and the microstructural characterization of these alloys in the as-cast condition [6,7,8,9,10].

**Figure 13:**
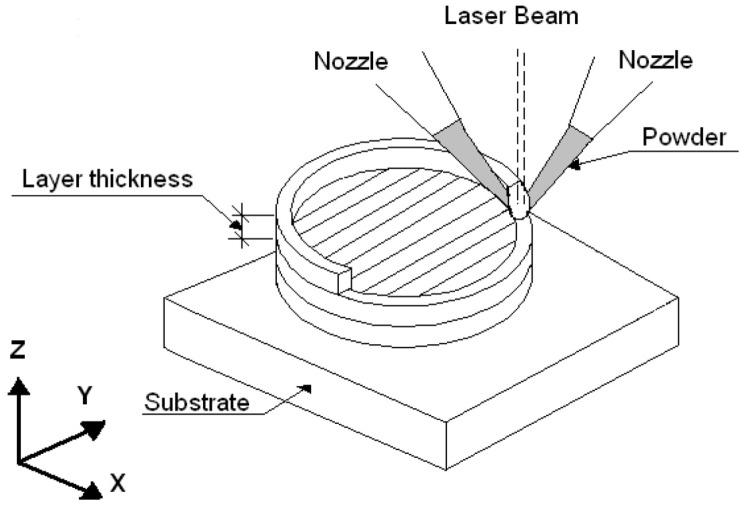
Schematic representation of the LENS process [24].

During laser forming combined with direct synthesis, the powders were fed into a melt pool that was produced by a sharply focused laser beam. The samples were built in a layer-by-layer fashion by rastering the laser and powder source across the substrate (Figure 13). The LENS processing parameters in the experiments were as follows: laser power of 250 W, laser spot diameter of 0.7 mm, scanning speed of 12 mm/s and thickness of the deposited layer of 0.25 mm. The complete deposition process was performed in a chamber under a continuous atmosphere of purified argon. The amounts of oxygen and water were less than 10 ppm. A commercial, pure iron (Armco supplied by Metal-Stal, Poland) plate with a thickness of 15 mm was used as the substrate for the depositions. All handling of the powders with the powder feeders was conducted in a Labmaster Glovebox Workstation (MBraun, Germany) under a continuous atmosphere of purified argon. The amounts of oxygen and water were less than 0.1 ppm. The LENS-deposited samples had a cylindrical geometry, with a diameter of 25 mm and a height of 3–6 mm. The as-deposited samples were sectioned using electro-discharge machining. One part of each deposited alloy was heat-treated for homogenization by annealing at 1000 °C for 20 h in an argon atmosphere with furnace cooling.

The morphology of the initial powders and the microstructures of the laser *in situ* deposited and heat-treated alloys were studied by optical light microscopy (Nikon MA 200 equipped with an image analyzer) and high-resolution scanning electron microscopy (SEM) using a Quanta 3D FEG Dual Beam (Fei, Hillsboro, OR, USA) equipped with a backscatter electron (BSE) detector, a duo-scanning transmission electron microscope (STEM) equipped with a bright/dark field (BF/DF) detector, a focused ion beam (FIB) and an orientation imaging microscope (OIM) system, which is based on backscattered electron diffraction. A liquid ion metal source was used as the source of the gallium ion beam for cutting the thin samples by FIB. Platinum was used as a protective layer.

The grain boundary misorientation angles of the investigated alloys were measured using OIM. Grain boundaries with angles below 15° were defined as low-angle boundaries (LABs), while grain boundaries with angles greater than 15° were defined as high-angle boundaries (HABs). The grain (phase) size measurements were performed using an automatic image analyzer attached to the OIM system. The parameters of the individual grains (phases) that were measured were A, which is the grain section area, and deq, which is the equivalent circle diameter of the grain (phase) (d_eq_ = [4A/π]^1/2^). A stereological method based on the percent area that was measured during the image analysis was performed on the as-polished deposits to characterize the amount of porosity.

The chemical compositions of the alloys after their direct laser deposition and subsequent heat treatment were established through wavelength dispersive X-ray fluorescence (XRF) spectroscopy measurements using a ZSX Primus II (Rigaku, Tokyo, Japan) that was equipped with a device for macro-mapping elements. The XRF measurements were performed in a circular area with a diameter of 25 mm on cross-sections of cylindrical samples. Additionally, energy dispersive X-ray spectroscopy (EDS) was performed using an EDAX system attached to a Quanta SEM (Fei, Hillsboro, OR, USA) to measure the chemical compositions of the microregions.

The XRD analysis was conducted with an Ultima IV (Rigaku, Tokyo, Japan) using CuKα radiation (λ = 1.5406 Å) and operating at 40 mA and 40 kV. The scan range was from 2θ = 20°–100° with a scan speed of 0.5°/min and a step size of 0.02°. Phase analysis was also conducted using OIM to establish the morphology and distribution of the identified phases in the microstructure. The Vickers microhardness was measured with a type M automated microhardness tester (Shimadzu, Kyoto, Japan) with a test load of 100 g and a dwell time of 5 s.

## 4. Conclusions

The LENS technique can be used for the direct synthesis of ternary L2_1_–ordered Fe-Al-Ti-based intermetallics from a feedstock composed of pre-alloyed Fe-Al powders and elemental Ti powder. The obtained average compositions of the ternary alloys measured by XRF after laser deposition and subsequent annealing were quite close to the nominal compositions. However, the XRF macro-maps recorded over a large area (5 × 5 mm) of the alloys revealed that the distributions of elements in the deposited and subsequently annealed samples were not fully homogeneous. Even after the high-temperature annealing, there were still some regions in the samples where the amounts of Al and Ti were essentially different from the average composition. No pure Ti was observed in the deposited alloys.

The macroscopic cracking caused some samples to delaminate from the substrate, and disintegration of the samples due to high thermal stress was occasionally observed. In addition, spherical pores within the deposited samples were observed in all the alloys. The amount of porosity in the deposits was less than 1.2 vol. %. Based on the pore morphology, it can be assumed that these porosities are due to the porous pre-alloyed Fe-Al powders.

The single-phase (L2_1_), two-phase (L2_1_-C14) and multiphase (L2_1_-A2-C14) Fe-Al-Ti intermetallic alloys were obtained after a direct laser synthesis and annealing process. The most prominent feature of the ternary Fe-Al-Ti intermetallics synthesized by LENS is their fine-grained structure after laser deposition. The grain size was 3–5 μm, which demonstrates the grain refinement effect through the very rapid cooling of the LENS process.

The ternary Fe-Al-Ti alloys annealed at 1000 °C in the single-phase B2 region were prone to an essential grain growth. In contrast, the alloys annealed at 1000 °C in the two-phase L2_1_-C14 region exhibited almost constant grain sizes after high-temperature annealing. This behavior can be attributed to the effect of the Laves phase precipitates, which effectively restrain the movement of grain boundaries.
